# Medial Plica Syndrome of the Knee: Arthroscopic Plica Resection versus Structured Physiotherapy—A Randomized Controlled Trial

**DOI:** 10.1055/s-0042-1756183

**Published:** 2022-09-19

**Authors:** Steffen Sauer, Gitte Karlsen, Lene Miller, Jens Ole Storm

**Affiliations:** 1 Aleris Hospital, Aarhus, Denmark; 2 Regional Hospital, Silkeborg, Denmark; 3 Aarhus University Hospital, Aarhus, Denmark

**Keywords:** knee, medial plica, plica syndrome

## Abstract

**Background** Medial plica syndrome is a commonly overlooked cause of anterior knee pain. A consensus on diagnosis and treatment is yet to be found. This study compares the clinical outcome of arthroscopic plica resection with structured physiotherapy for patients with isolated medial plica syndrome in a prospective randomized controlled trial with a 2-year follow-up.

**Methods** Forty-eight patients have been included in this prospective randomized controlled trial presenting medial plica syndrome. Patients were randomly assigned to either arthroscopic plica resection or structured physiotherapy. The primary outcome was the change in the average score of the Lysholm knee scoring scale from baseline to 2-year follow-up.

**Results** Mean baseline Lysholm score for patients assigned to arthroscopic plica resection and physiotherapy was 65.8 and 66.3, respectively. No significant difference was seen between the two groups. Thirty-three patients were assessed at 2 years follow up. The mean Lysholm score was 89.7 for patients assigned to arthroscopic plica resection and 74.6 for patients assigned to structured physiotherapy. A statistically significant difference was seen between the two groups (p = 0.007).

**Conclusions** Arthroscopic plica resection was associated with significantly greater clinical improvement compared with physiotherapy at 2-year follow-up.

Medial plica syndrome of the knee represents a commonly overlooked cause of anterior knee pain. 1 2 While the pathological potential of medial plicae is widely agreed upon, the incidence of plica syndrome remains unknown, mostly because diagnostic criteria are lacking. Estimates for the frequency of plica syndrome based upon small arthroscopic series of patients presenting with acute knee pain range from 3 to 30%. 2 Pathological plicae may develop after blunt trauma or mechanical irritation due to repetitive knee movements that cause inflammation of the plica with subsequent fibrosis. 1 3 The resulting thickening and alteration of elasticity cause the plica to snap over the medial femoral condyle causing synovitis, chondral damage, and pain. 4 However, direct contact between plicae and the patellofemoral joint is not necessary to cause symptoms as the tethering effect of a thickened plica might interfere with normal quadriceps function, placing excessive traction on its richly innervated synovial insertion. 5 Findings of medial plica syndrome are unspecific and limited. Tenderness on direct palpation over the anteromedial aspect of the capsule is frequently present. Furthermore, a thickened cord-like band may be present, in some cases producing a noticeable snap. 3 6 A variety of provocation tests have been described, mostly based on simulating provocative conditions that will aggravate symptoms. 4 7 8 The goals of treatment for plica syndrome are to reduce pain and return the patient to a high level of function. Current treatment algorithms are based on physicians' experience and a primum non nocere philosophy rather than rigorous evidence. No randomized controlled trial has ever elucidated the effect of physiotherapy in the treatment of medial plica syndrome. The presumed positive effect of physiotherapy is mostly derived from reports like by Camanho titled “Treatment of pathological synovial plicae of the knee.” 9 The conclusion that surgical treatment should be reserved for exceptional cases is drawn from the fact that 78% of patients were free of symptoms after undergoing conservative treatment involving strengthening and improved flexibility of muscles surrounding the knee. Arthroscopic plica excision commonly shows good clinical results as reported by Schindler in the meta-analysis of the Sneaky Plica, which identifies 23 studies assessing the clinical outcome of 969 patients following open or arthroscopic plica excision. 10 These studies are, however, substantially inhomogeneous with different inclusion criteria, outcome measures, follow-up periods, as well as surgical techniques.

The purpose of this prospective randomized controlled trial was to evaluate the effect of structured physiotherapy in comparison to arthroscopic plica resection for patients with medial plica syndrome. We hypothesized that physiotherapy would be significantly superior to arthroscopic plica resection regarding symptom remission.

## Materials and Methods

### Trial Design

The present trial was conducted as a prospective randomized controlled multicenter trial comprising four hospitals. Based on an a priori statistical power calculation for sample size, a total of 40 patients diagnosed with medial plica syndrome was intended to be enrolled in the trial. The primary outcome was defined as the change in the Lysholm score between baseline and 2-year follow-up. The mandatory time of physiotherapy was limited to 3 months to facilitate patient recruitment and avoid selection bias. Patients were strongly advised to continue with the training-protocol as long as possible and it was assumed that patients would continue with self-conducted training as necessary over a period of minimum 2 years. Follow-up appointments were chosen after 3 and 6 months to evaluate short-term effects of both treatments as well as after 1 and 2 years to evaluate the effect of self-conducted training and eventual long-term surgical failures related to plica recurrence. It was expected that physiotherapy would result in a significantly higher Lysholm score compared with arthroscopic plica resection at 2-year follow-up. The trial protocol was registered at ClinicalTrials.gov prior to trial inception (NCT02578589NC). Following approval by the local ethics committee (ref. 1–6,9–14–72–360–13), a declaration of consent was signed by patients willing to participate in the trial and adequate time for contemplation was given. Patients were informed both orally and in writing about treatment options and trial participation modalities. Conditions regarding patient recruitment, inclusion, randomization, treatment algorithms, surgical technique, training protocols as well as follow-up were identical in all hospitals.

### Subjects

The subject population consisted of patients with anteromedial knee pain in which differential diagnoses were excluded by means of magnetic resonance imaging (MRI) and where plica-syndrome was clinically suspected. Inclusion criteria were as follows:

### Inclusion Criteria

Patients were invited to participate in the trial if they fulfilled all of the following criteria: Patient aged over 18 years, history of focal anteromedial knee pain, medial plica appearance on MRI, absence of other intra- and extra-articular pathology as well as given declaration of consent. The presence of focal anteromedial knee pain was mandatory; patients with concentric, diffuse pain around the patella were not included.

### Exclusion Criteria

Patients were excluded from the trial if they fulfilled one of the following criteria:

Presence of any intra- or extra-articular pathology including the following diagnoses or conditions: osteoarthritis, osteochondritis dissecans, meniscal lesion, patella luxation, subluxation or instability, pes anserinus bursitis, diffuse pain around the patella suggesting patellofemoral pain syndrome, prior surgical intervention, or infection of the relevant knee.

### Procedure

If inclusion criteria were applicable, patient history and physical examination findings including unspecific criteria of plica-syndrome were assessed and recorded. Patients were asked to fill out the following questionnaires: Lysholm knee scoring scale, Kujala anterior knee pain score, visual analog scale (VAS)-pain-score while resting, during light and heavy physical activity as well as the quality-of-life (QOL) subscale extracted from KOOS (knee injury and osteoarthritis outcome score).

**Fig. 1:**
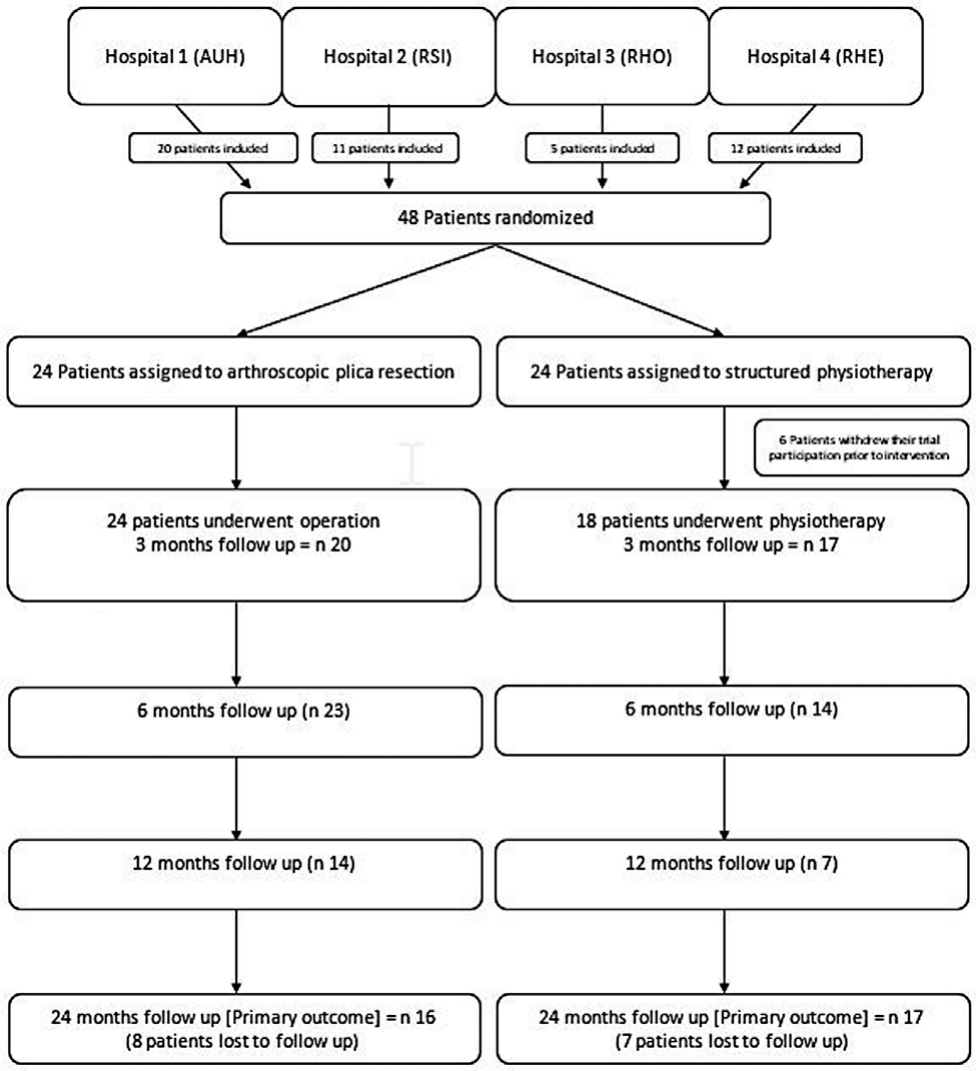
Medial plica syndrome of the knee: arthroscopic plica resection versus structured physiotherapy—randomized controlled trial CONSORT flowchart.

Forty-eight patients were included in this trial between September 2014 and December 2017 presenting with isolated clinically diagnosed medial plica syndrome. MRI showed a concurrent medial plica in all cases and no other structural pathology. Patients were randomly allocated to subject-group 1 (arthroscopic medial plica resection) or subject-group 2 (conservative treatment)( Fig. 1 ).

### Arthroscopic Medial Plica Resection (Patient-Group 1)

Arthroscopic medial plica resection was performed by experienced surgeons under general anesthesia in all cases. All joint compartments of the knee were thoroughly assessed and any concomitant intra-articular pathology if present was planned to be addressed prior to resection of symptomatic plicae. Pathological plicae were resected entirely with a radiofrequency device, strictly avoiding capsular damage, thus avoiding postoperative hemarthrosis. Eventual supra- and infrapatellar plicae were left in situ. The intervention was performed as a day case procedure; early range of motion exercises 3 to 4 days postoperatively were encouraged to prevent intra-articular scarring and subsequent stiffness. Patients were given an unspecific standardized postarthroscopy mobilizing program and oral analgesic medication. Immediate postoperative weight bearing was permitted. Sutures were removed 12 days postoperatively by the patient's general practitioner.

### Physiotherapy (Patient-Group 2)

All patients underwent the same structured rehabilitation program for a minimum period of 3 months. The rehabilitation program (available online at plicaprojekt.com) was created by the authors in accordance with current literature and in collaboration with experienced sports physiotherapists. The exercises focused on strengthening of the quadriceps muscles as well as hip stabilization. The rehabilitation program included the following exercises for strengthening quadriceps muscles: quadriceps set exercise, straight leg raise exercise, leg press exercise, stationary bike exercise, and the use of an elliptical machine. Concurrent with this, patients worked on a frequent stretching program including hamstrings, mm. adductores, m. gastrocnemius, and m. quadriceps. Physical activity inducing plica irritation was advised against. All open chain exercises (foot is free to move) that eventually cause plica irritation were avoided. Regarding adjunctive therapy, none of the patients received biomechanical realignment procedures or intra-articular steroid injections. On initiation of the rehabilitation program, patients were assessed by a physiotherapist on a weekly basis for the period of 4 weeks. Patients were asked to demonstrate correct stretching of knee flexor and extensor muscles as well as correct performance of strengthening exercises. Any issues concerning quality of performance were assessed and corrected. The rehabilitation program was modified in rare cases if symptom aggravation occurred and consisted in omitting the exercises that had led to symptom aggravation. The correct performance of the rehabilitation program as a whole without symptom aggravation was the criterion for release into fully self-conducted training, expected at 4 weeks after inception of the rehabilitation program.

### Follow-Up

Follow-up was performed independently of group allocation for all patients after 3 months, 6 months, 1 year, and 2 years after the date of their trial inclusion. At follow-ups, patients were assessed by an orthopaedic surgeon performing a standard knee examination to assess eventual complications. Patients were asked to fill out the following questionnaires: Lysholm knee scoring scale, Kujala anterior knee pain score, VAS score, and QOL (extracted from KOOS).

### Statistics and Sample Size

Data are presented as means ± standard error and were analyzed using SPSS (SPSS, Inc., Chicago IL). Differences in outcomes were assessed by Student's *t* -test. The level of statistical significance was set to *p* < 0.05. A priori sample size was calculated based on the assumption that patients undergoing conservative treatment would reach a 10-point higher mean value on the Lysholm knee scoring scale at 2-year follow-up compared with patients undergoing arthroscopic plica resection. The mean Lysholm score in normal knees was assumed to be 93.4 (standard deviation: 8.87; min 57, max 100) as described by Anderson et al. 11 The population value was 0.8 (80/100 points) as for the expected mean Lysholm score regarding arthroscopic plica resection and 0.9 (90/100 points) as for the expected mean Lysholm score regarding physiotherapy. A two-sided test (α 0.05, power 0.8, and delta 8.87 (standard deviation) indicated a required sample size of 16 patients for each group. The dropout rate was expected to be 20%. Thus, a minimum of 40 patients were intended to be included in this trial.

## Results

### Demographics and Anamnestic Information

Forty-eight patients were included in this trial. Patient-group 1 (arthroscopic plica resection) comprised 24 patients (of these, nineteen female and five male) with a mean age of 27 years and a mean symptom duration of 23.6 months. Group 2 (physiotherapy) comprised 24 patients (of these, fourteen female and ten male) with a mean age of 26.6 years and a mean symptom duration of 26 months. No significant difference was seen between the two groups. Plica-related anamnestic details are summarized in Table 1 . In addition to focal medial parapatellar pain (inclusion criteria), patients most commonly experienced a subjective click-sensation and pain while sitting. A history of trauma was only reported in 16% of the cases; swelling was only reported in 8% of the cases.

**Table 1:** Incidence of plica related anamnestic details

*n* = 48	Percent
History of trauma	16
Click-sensation	67
Instability	25
Pain while sitting	67
Snapping sensation	25
Swelling	8
Suprapatellar pain	42
Infrapatellar pain	58

### Arthroscopy

Twenty-four patients underwent arthroscopic plica resection. A mediopatellar plica was found and resected in all cases. In all patients, photo documentation of the medial plica as well as the patellofemoral, medial and lateral knee joint compartment was performed. None of these patients showed other relevant intra-articular pathology that required treatment. Most patients presented a type A or B medial plica (according to Sakakibara) that corresponds to a chord like elevation or a wider shelf. 12 No surgical complications were seen.

### Physiotherapy

Eighteen patients underwent the given physiotherapy protocol (available online at plicaprojekt.com). Nine patients completed training diaries that all showed continuous increase of training intensity. Most patients were able to perform the protocol without symptom aggravation and subsequent program modification. No complications regarding the execution of the training program were seen.

### Crossover

From baseline to 2 years follow-up, one patient who had been assigned to structured physiotherapy underwent arthroscopic plica resection; the structured trainings program was hereby not initiated. None of the patients who underwent arthroscopic plica resection performed the given or a similar structured physiotherapy protocol postoperatively.

### Lysholm (Primary Outcome)

**Fig. 2:**
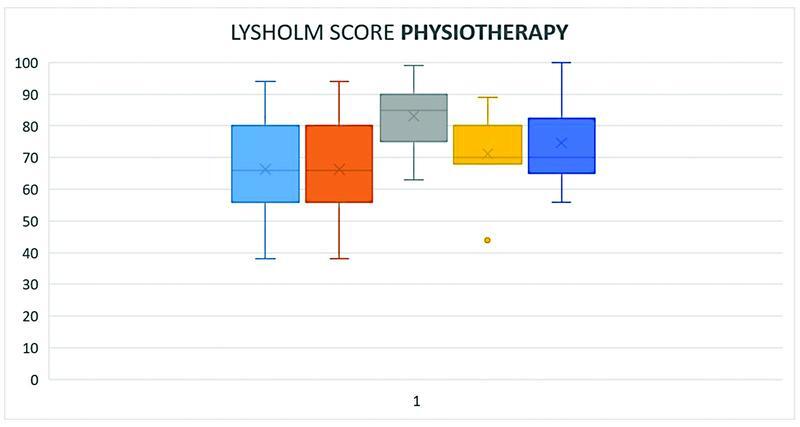
Boxplot diagram showing min and max, mean and median as well as percentiles of the Lysholm score of patients who underwent physiotherapy at baseline and follow-up.

**Fig. 3:**
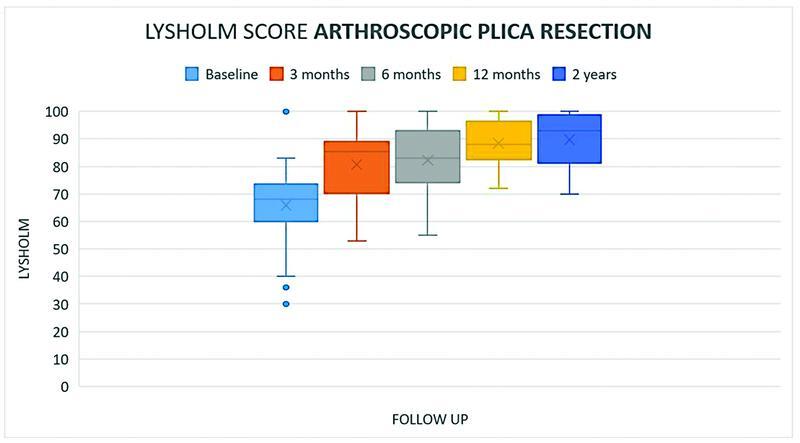
Boxplot diagram showing min and max, mean and median as well as percentiles of the Lysholm score of patients who underwent arthroscopic plica resection at baseline and follow-up.

**Fig. 4:**
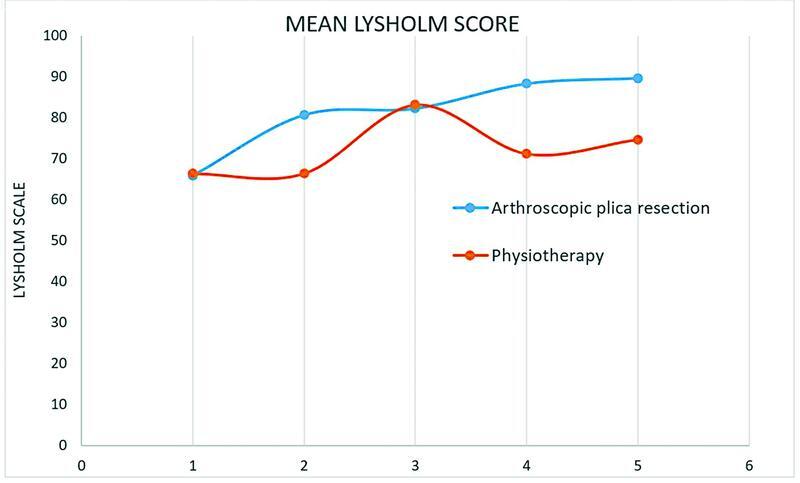
Mean Lysholm score of arthroscopic plica resection (blue) and physiotherapy (orange) at baseline (1) and 3-month follow-up (2), 6-month follow-up (3), 1-year follow-up (4), and 2-year follow-up (5).

Baseline mean Lysholm score was 65.8 points for patients assigned to arthroscopic plica resection and 66.3 points for patients assigned to structured physiotherapy. No significant difference was seen between the two groups. At 3-month follow-up, mean Lysholm score was 80.7 points for patients assigned to arthroscopic plica resection and 74.9 points for patients assigned to structured physiotherapy. The difference between the groups was not significant. Thirty-eight patients were assessed at 3-month follow-up. Six patients withdrew their trial participation, and five patients were lost to follow-up. At 6-month follow-up, the mean Lysholm was 82.3 points for patients assigned to arthroscopic plica resection and 83.1 points for patients assigned to structured physiotherapy. The difference between the groups was not significant. Both groups exceeded to minimal clinical important difference of 10.1 points from baseline to 6-month follow-up. Thirty-seven patients were assessed at 6-month follow-up. At 1 year follow-up, the mean Lysholm score was 88.3 for patients assigned to arthroscopic plica resection and 71.1 points for patients assigned to structured physiotherapy. The difference between the groups was not significant. Only 21 patients were assessed at 1 year follow-up due to a failure to invite patients to the given follow-up. At 2 years follow-up, the mean Lysholm score was 89.7 for patients assigned to arthroscopic plica resection and 74.6 for patients assigned to structured physiotherapy. A statistically significant difference was seen between the two groups ( *p* = 0.007). Thirty-three patients were assessed at 2 years follow-up, which corresponds to a follow-up rate of 68% for the primary outcome (Lysholm)( Figs. 2 Fig. 3 Fig. 4 ; Table 2 )

**Table 2:** Mean Lysholm score and±standard error for patients that underwent arthroscopic plica resection and physiotherapy at baseline and follow-ups

Lysholm	Arthroscopic plica resection	Physiotherapy	Significance
Baseline ( *n=* 48)	65.8±3.2	66.3±3.3	ns
3 months ( *n* =38)	80.7±3.3	74.9±4.1	ns
6 months ( *n* =37)	82.3±2.6	83.1±2.6	ns
1 year ( *n* =21)	88.3±2.5	71.1±5.4	ns.
2 years ( *n* =33)	89.7±2.3 ( *n* =16)	74.6±3.2 ( *n* =17)	*p* =0.007

Abbreviation: ns, not significant.

### Kujala

Baseline mean Kujala score was 69.4 points for patients assigned to arthroscopic plica resection and 70.6 points for patients assigned to structured physiotherapy. No significant difference was seen between the two groups. At 3-month follow-up, mean Kujala score was 82.7 for patients assigned to arthroscopic plica resection and 73.1 for patients assigned to physiotherapy; the difference between groups was not significant. At 6-month follow-up, mean Kujala score was 85.2 for patients assigned to arthroscopic plica resection and 80.6 for patients assigned to physiotherapy; the difference between groups was not significant. At 1 year follow-up, mean Kujala score was 87.4 for patients assigned to arthroscopic plica resection and 76.3 for patients assigned to physiotherapy; the difference between groups was not significant. At 2 years follow-up, mean Kujala score was 93.0 for patients assigned to arthroscopic plica resection and 74.1 for patients assigned to physiotherapy. A statistically significant difference between the two groups was seen ( *p* <0.001). Thirty-three patients were assessed at 2 years follow-up. Twelve of these patients omitted secondary outcome questionnaires (Kujala, VAS, QOL), which had led to lower responds number (n21) compared with the primary outcome questionnaire (Lysholm).( Table 3 ).

**Table 3:** Mean Kujala score and±standard error for patients that underwent arthroscopic plica resection and physiotherapy at baseline and follow-ups

Kujala	Arthroscopic plica resection	Physiotherapy	Significance
Baseline ( *n* =48)	69.4±3.1	70.6±3.1	ns
3 months	82.7±2.9	73.1±5.2	ns
6 months	85.2±2.4	80.6±2.7	ns
1 year	87.4±2.6	76.3±5.2	ns
2 years ( *n* =21)	93.0±2.3 ( *n* =12)	74.1±4.0 ( *n* =9)	*p* =0.001

Abbreviation: ns, not significant.

### Visual Analog Scale

Baseline mean VAS score for patients assigned to arthroscopic plica resection under rest, walking, and physical activity was 2.5, 3.3, and 7.0, respectively. For patients assigned to physiotherapy, mean baseline VAS score under rest, walking, and physical activity was 2.6, 3.1, and 7.0, respectively. Regarding VAS scores, no significant difference was seen between the groups. At 3-month follow-up, mean VAS score for patients assigned to arthroscopic plica resection under rest, walking, and physical activity was 2.6, 1.7, and 3.2, respectively. For patients assigned to physiotherapy, mean baseline VAS score under rest, walking, and physical activity was 2.6, 2.8, and 4.9, respectively. No significant difference was seen between the groups. At 6-month follow-up, mean VAS score for patients assigned to arthroscopic plica resection under rest, walking, and physical activity was 1.2, 1.8, and 3.4, respectively. For patients assigned to physiotherapy, mean baseline VAS score under rest, walking, and physical activity was 1.5, 2.3, and 4.1, respectively. No significant difference was seen between the groups. At 1 year follow-up, mean VAS score for patients assigned to arthroscopic plica resection under rest, walking, and physical activity was 0.5, 0.9, and 2.1, respectively. For patients assigned to physiotherapy, mean baseline VAS score under rest, walking, and physical activity was 1.6, 3.0, and 3.7, respectively. No significant difference was seen between the groups. At 2 years follow-up, mean VAS score for patients assigned to arthroscopic plica resection under rest, walking, and physical activity was 0.4, 0.9, and 2.5, respectively. For patients assigned to physiotherapy, mean baseline VAS score under rest, walking, and physical activity was 3.1, 3.4, and 5.7, respectively( Table 4 ).

**Table 4:** Mean VAS score and±standard error for patients that underwent arthroscopic plica resection and physiotherapy at baseline and follow-ups

VAS	Arthroscopic plica resection			Physiotherapy		
Intensity	Rest	Walk	Activity	Rest	Walk	Activity
Baseline	2.5±0.4	3.3±0.5	7.0±0.5	2.6±0.5	3.7±0.4	7.0±0.4
3 months	2.6±0.4	1.7±0.4	3.2±0.5	2.6±0.9	2.8±0.7	4.9±1.0
6 months	1.2±0.3	1.8±0.4	3.4±0.5	1.5±0.6	2.3±0.8	4.1±0.7
1 year	0.5±0.4	0.9±0.3	2.±0.5	1.6±0.8	3.0±0.9	3.7±0.7
2 years	0.4±0.1	0.9±0.3	2.6±0.7	3.1±1.2	3.4±0.9	5.7±1.1

Abbreviation: VAS, visual analog scale.

### Quality of Life

Patients assigned to arthroscopic plica resection showed a significant higher baseline QOL score with 53 points in comparison to patients assigned to physiotherapy with 40 points ( *p* =0.009). At 3-month follow-up, mean QOL was 55 for patients assigned to arthroscopic plica resection and 50.5 for patients assigned to physiotherapy. At 6-month follow-up, mean QOL was 65 for patients assigned to arthroscopic plica resection and 59.4 for patients assigned to structured physiotherapy. At 1 year follow-up, mean QOL was 69 for patients assigned to arthroscopic plica resection and 55 points for patients assigned to structured physiotherapy. At 2 years follow-up, mean QOL was 78 for patients assigned to arthroscopic plica resection and 44.4 points for patients assigned to structured physiotherapy( Table 5 ).

**Table 5:** Mean QOL score and±standard error for patients that underwent arthroscopic plica resection and physiotherapy at baseline and follow-ups

QOL	Arthroscopic plica resection	Physiotherapy	Significance
Baseline	56.0±3.1	41.7±3.5	*p* =0.009
3 months	55.0±3.8	50.5±5.1	ns
6 months	65.0±3.8	59.4±4.6	ns
1 year	69.0±8.8	50.0±5.7	ns
2 years	78.0±4.7	44.4±6.4	*p* =0.001

Abbreviations: ns, not significant; QOL, quality of life.

## Discussion

The primary finding of this trial was that patients who underwent arthroscopic plica resection showed a significantly higher Lysholm score compared with patients who underwent physiotherapy at 2 years follow-up. The initial hypothesis that physiotherapy would be superior to arthroscopic plica resection at 2 years follow-up was not confirmed. However, both arthroscopic plica resection and physiotherapy resulted in comparable and significant short-term clinical improvement without between group differences at 6-month follow-up. In the following, patients treated operatively showed continuous clinical improvement, while patients treated conservatively showed clinical deterioration. The conclusion is conceivable that the positive effect of physiotherapy is bound to its continuous execution and that deterioration of symptoms is likely to occur, once the continuous and disciplined execution of the trainings protocol is abandoned. The combination of low symptom recurrence after arthroscopic plica resection and low compliance to self-conducted physiotherapy may be the reason for the significant disparity between the two groups concerning the Lysholm score after 2 years.

The surgical results of this trial are in accordance with Schindler's meta-analysis of 23 studies assessing the clinical outcome of 969 patients following open or arthroscopic plica excision. 10 On the whole, good-to-excellent results are shown with 64% of the patients being free of symptoms, 26% having improved outcome, and 10% being considered failures.

Very few studies have examined the outcome of conservative treatment of patients with plica syndrome, one of the most relevant being treatment of pathological synovial plicae of the knee. 9 13 The conclusion that surgical treatment should be reserved for exceptional cases is hereby drawn from the fact that 78% of patients were free of symptoms after undergoing conservative treatment involving strengthening and improving the flexibility of muscles surrounding the knee. However, the report by Camanho 9 does not rigorously differentiate between medial and suprapatellar plicae. Furthermore, the real incidence of plica syndrome remains unknown due to the absence of a surgical control group.

To minimize the risk of confounding with concurrent disorders, patients were only included in this trial if they presented with focal and locatable medial parapatellar pain. Patients with diffuse pain around the patella were not included due to a potential clinical overlap with pure patellofemoral disorders.

The minimum period of assisted physiotherapy was predefined to 3 months. A longer course could have been desirable but would eventually have impeded patient recruitment. Furthermore, the risk of selection bias would have increased as only highly motivated patients would have agreed to participate in the trial that may have led to a better training outcome of our trial population compared with the normal population. Patients were strongly advised to continue the trainings protocol as necessary until the final follow-up appointment 2 years after the protocol was commenced. Even though not objectively and systematically recorded, the majority of patients declared that they abandoned the trainings-protocol after 3 to 6 months.

Numerous studies have elucidated plica syndrome and emphasized a variety of accompanying challenges concerning study design leading to an inhomogeneous body of research. 2 15 16 Among these challenges roam predominantly that clinical criteria and imaging methods for making the diagnosis are lacking. As plica syndrome represents an exclusion diagnosis, the exact number of eligible patients may not easily be identified: patients suffering from plica syndrome are rarely referred to specialist with the diagnosis plica syndrome and patients that were referred with plica syndrome as the working diagnosis did rarely suffer from plica syndrome. Furthermore, eligible patients with concomitant but potentially asymptomatic pathology (e.g., degenerative meniscus injury) were not identified as eligible patients. Thus, the real number of eligible patients remains unknown. The resulting potential selection bias may lead to an overrepresentation of highly motivated patients with a better outcome regarding physiotherapy compared with the normal population. No significant difference was seen between the trial population and the population unwilling to participate in the trial concerning age and symptom duration. The results of this trial indicate that focal medial anterolateral pain may be considered as the cardinal symptom of plica syndrome. Out of 23 patients who presented with focal pain and who subsequently underwent arthroscopic plica resection, all patients showed a fibrotic plica without significant concomitant pathology. Furthermore, a mean Lysholm score of approximately 90 points at 2 years follow-up following plica resection as the only surgical intervention supports plica syndrome as the correct diagnosis.

Our trial has certain limitations. The gold standard for the diagnosis plica syndrome remains arthroscopic evaluation, thus the exact number of pathological plicae and the incidence of concomitant intra-articular lesions in the conservative group remain unknown. Even though arthroscopically assessed, the visual interpretation of plicae in the surgical group was subjective, as not histologically verified. Especially regarding Sakakibara type A plicae, normal plicae could have been mistaken for pathological plicae. Furthermore, the impact of plica type on treatment outcome remains unknown. The diameter of a medial has been shown to be associated with unsuccessful conservative treatment of medial plica syndrome. 17 Further studies are needed to elucidate treatment algorithm and outcome based on plica morphology. In this trial, a minimum period of 3 months of physiotherapy was predetermined, along with a relatively long follow-up period of 2 years. Even though a longer period of mandatory physiotherapy would have been of interest, it would have impeded patient recruitment and increased the risk of selection bias. Furthermore, it was assumed that patients would continue with the trainings-protocol until symptom remission occurred and that patients would resume the trainings-protocol if necessary. The results of this trial may be biased by the fact that unsatisfied patients suffering from symptom recurrence following arthroscopic plica resection may have been less likely to participate in the 2-year follow-up appointment compared with unsatisfied patients following physiotherapy, eventually aiming at crossover. This may have led to an overrepresentation of satisfied patients following surgery with a relatively higher Lysholm score. Regarding the physiotherapy group, six patients withdrew their trial participation prior to the inception of physiotherapy, eventually leading to an overrepresentation of highly motivated patients in this group. The fact that only one patient was afterward lost to follow-up in the physiotherapy group may underline this assumption, which could have led to a higher mean Lysholm score than the average-motivated patient would have achieved. The exact decline regarding training intensity of patients in the physiotherapy group could not be rigorously quantified and remains unknown. Even though unlikely, it can therefore not be ruled out that the positive effect of physiotherapy is limited to a period of 6 months regardless of further optimal execution of a given trainings protocol. Finally, the effect of imbalanced female preponderance in both groups is unclear.

The strengths of this trial are its rigorous inclusion criteria and a reproducible and effective physiotherapy program. These aspects may facilitate diagnosis, assessment, and treatment of plica syndrome in the future. The fact that patients with plica syndrome show a low QOL score emphasizes the importance of awareness toward plica syndrome that remains an underdiagnosed cause of anterior knee pain and which has significant impact on QOL.

In conclusion, the results of this trial indicate that plica syndrome may be treated effectively by means of physiotherapy. However, high compliance to continuous self-conducted training may be of paramount importance for satisfactory long-term results. Surgery shows good results and should be taken into consideration in cases in which conservative measures fail or a lack of compliance to a given trainings protocol is assumed. Further studies are needed to investigate the effect of different training protocols and conservative approaches, eventually focusing on the possible impact of biomechanical alterations such as pelvic stabilization, iliotibial band elasticity, and dynamic valgus phenomenon.
